# Exceptional Bluetongue virus (BTV) and Epizootic hemorrhagic disease virus (EHDV) circulation in France in 2023

**DOI:** 10.1016/j.virusres.2024.199489

**Published:** 2024-11-01

**Authors:** Mathilde Gondard, Lydie Postic, Emmanuel Garin, Mathilde Turpaud, Fabien Vorimore, David Ngwa-Mbot, Mai-Lan Tran, Bernd Hoffmann, Charlotte Warembourg, Giovanni Savini, Alessio Lorusso, Maurilia Marcacci, Arnaud Felten, Aurélie Le Roux, Yannick Blanchard, Stephan Zientara, Damien Vitour, Corinne Sailleau, Emmanuel Bréard

**Affiliations:** a ANSES/INRAE/ENVA-UPEC, UMR 1161 Virology, Laboratoire de santé animale, 14 rue Pierre et Marie Curie, 94700 Maisons-Alfort, France; b Groupements de Défense Sanitaire France, 37 rue de Lyon, 75012 Paris, France; c Genomics Platform IdentyPath, Laboratory for Food Safety, ANSES, 94700 Maisons-Alfort, France; d Friedrich-Loeffler-Institut, Institute of Diagnostic Virology, Südufer 10, 17493 Greifswald - Insel Riems; e Société Nationale des Groupements Techniques Vétérinaires, 5 rue Moufle, 75011 Paris, France; f Istituto Zooprofilattico Sperimentale dell'Abruzzo e del Molise, Teramo, Italy; g Laboratory of Ploufragan, ANSES, Unit of Viral Genetics and Biosafety, Ploufragan, France

**Keywords:** Emergences, Bluetongue virus, Epizootic hemorrhagic disease virus, France, genome sequencing, nanopore

## Abstract

Bluetongue (BT) and Epizootic Hemorrhagic Disease (EHD) are two notifiable animal diseases transmitted to ruminants by small hematophagous midges belonging to the *Culicoides* genus. The etiological agents, Bluetongue virus (BTV) and Epizootic hemorrhagic disease virus (EHDV), are both members of the *Sedoreoviridae* family and *Orbivirus* genus, which include double-stranded (ds) RNA segmented genomes (10 segments). By the end of the summer 2023, first's outbreaks of EHD were reported from the south west of France, concurrently with unexpectedly severe BT cases in Central France and Corsica. Within a few weeks, numerous BT and EHD outbreaks were recorded with significant sanitary and economic impact on cattle and sheep farms (no sanitary impact of EHD for sheep). Using a customized SISPA approach and the nanopore sequencing technology we successfully recovered genomic sequences from viral isolates and blood samples from infected animals from EHD and BT outbreaks. Three different viruses were responsible for these outbreaks: EHDV-8, BTV-8 and BTV-4. The EHDV-8 strain detected in France corresponded to the strain circulating in Tunisia, Sardinia and Spain since 2021 and 2022. A new BTV-8 strain of unknown origin, clearly different from the enzootic strain circulating in France since 2015, was responsible of the BT outbreaks in domestic ruminants in 2023 on both mainland France and Corsica. A second BTV, BTV-4, also involved in BT outbreaks in Corsica, corresponded to a BTV-4 strain occasionally detected on Corsica island since 2016, suggesting either a new introduction of this strain or a silent circulation on the field. The exceptional nature of orbivirus epizootics in France in 2023, including new introduction, emergence or incursions, raises numerous questions regarding BTV and EHDV dynamics and epidemiology and stresses out the need to identify factors involved in these emergences.

## Highlights

• First introduction of EHDV-8 in France followed by EHD outbreaks in cattle.
• Emergence of a new BTV-8 strain in France responsible of severe BT outbreaks.
• Successful viral genome sequencing from blood samples using Nanopore technology.
• Risk of co-circulation of various BTV genotypes and viral reassortment.

## 1 Introduction

BT and EHD are two vector-borne diseases of wild and domestic ruminants listed by the World Organization for Animal Health (WOAH) as notifiable diseases since 2008. The two viruses responsible of these diseases, BTV and EHDV, are members of the genus *Orbivirus* of the *Sedoreoviridae* family, which have a non-enveloped capsid and a segmented dsRNA genome (10 segments) (Maclachlan et al., 2019).

Both arboviruses are mainly transmitted to animals through bites of female hematophagous midges belonging to the *Culicoides* genus, a vector encountered in most parts of the world (Harrup et al., 2015; Sick et al., 2019). Although BTV and EHDV can infect similar host species among ruminants, presence of clinical manifestations or severity of the infection can greatly vary according to the viral species and the strain (Jiménez-Cabello et al., 2023). BT is associated with economic losses in the livestock industry, especially in naïve animal populations. Whereas milder clinical symptoms can be observed in cattle or goats, which can act as reservoir of the pathogen, severe clinical signs are more often reported in sheep, with hemorrhages and inflammations resulting from vascular endothelial damages. Clinical signs includes edema of the head, swelling of the tongue, and inflammation of the coronary bands (Maclachlan et al., 2015a). EHD was mainly reported as a fulminant hemorrhagic disease in white-tailed deer, yet some serotypes can induced BT-like symptoms in cattle, which can lead to fatal cases (Maclachlan et al., 2015b; Sghaier et al., 2022). Similarly to BT, EHD is responsible for significant economic losses, affecting greatly the livestock industry when introduced in EHDV-free country and naïve bovine population.

BTV and EHDV genomes share the same organization, including 10 dsRNA segments (S) encoding 7 structural viral proteins (VP), called VP1 to VP7 and 6 non-structural (NS) proteins referred as NS1, 2, 3, 3a, 4, and 5, involved in viral replication cycle and host antiviral response escape mechanisms (Anthony et al., 2009a; Ratinier et al., 2016; Roy, 2005; Stewart et al., 2015). VP2 (S2) and VP5 (S6) form the outer capsid which contains major virus neutralization epitopes inducing the production of serotype-specific neutralizing antibodies. They are therefore used for virus serotype determination (Anthony et al., 2009b; Maan et al., 2007). VP7 (S7) and VP3 (S3) form the inner capsid containing the viral genome (one copy of each segment) and the replication complex involved in genome replication, namely VP1 (S1), the RNA-dependent RNA polymerase, VP4 (S4), and VP6 (S9, bicistronic).

To date, up to 36 BTV serotypes have been described including 24 traditional BTV serotypes (BTV-1–24), presenting a classical enzootic cycle involving vertebrate and invertebrate hosts, and more atypical serotypes (BTV-25–36) mainly reported in healthy goats and sheep in different countries (Bumbarov et al., 2020; Hofmann et al., 2008; Maan et al., 2011; Ries et al., 2020; Savini et al., 2017; Schulz et al., 2016; Sun et al., 2016; Wright, 2014). For a long time, BTV were thought to be restricted to tropical and subtropical regions, between 40° and 50° North latitude and between 20° and 30° South latitude. However, since the end of the 20th century, multiple outbreaks of BT occurred in European countries with BTV strains suspected to mainly follow the same path, from North Africa to the South of Europe (Kundlacz et al., 2019). Finally, BTV is considered as enzootic in several European countries such as France. On mainland France, an enzootic BTV-8 strain is circulating since 2015, and some BTV-4 strains were rarely detected since 2017. In Corsica, two strains of BTV-4 were reported since 2016.

Seven EHDV serotypes have been described worldwide, called EHDV-1 (including the IbAr 22619 strain formerly called EHDV-3), EHDV-2, 4 to 8 (Savini et al., 2011). Recently, a new member of the group, tentatively named “EHDV-10″ has been first detected and isolated from bovine blood in Japan and then from *Culicoides* in China (He et al., 2024; Shirafuji et al., 2017). Until 2022, Europe was free from EHDV. However, several EHDV strains were known to circulate since 2000s within North African countries including EHDV-1, 2, 6 and 7 (Golender and Hoffmann, 2024; Jiménez-Cabello et al., 2023; Sghaier et al., 2022). In 2021, the identification of EHDV-8 during an outbreak in cattle from Tunisia was unexpected as this serotype had only been reported in Australia in 1982 (Sghaier et al., 2022). EHDV-8 emerged in Europe in 2022, first reported in symptomatic cattle from Sardinia and Sicily islands, and later in Spain (Jiménez-Cabello et al., 2023; Lorusso et al., 2023). From Spain, EHDV-8 started its rise towards the north during summer 2023.

In September 2023, EHDV-8 crossed the border leading to multiple outbreaks in cattle in the south-west of France. In parallel, atypical severe BT cases linked to serotype 8 were observed in sheep and cattle in the southern Massif Central (Aveyron and neighboring departments) and later in Corsica (October 2023). Finally, BTV-4 outbreaks were also reported in sheep in Corsica. In this study, we present the preliminary epidemiological data on BT and EHD impact in France in 2023, based on field surveys organized by the local breeder association networks (Groupement de Défense Sanitaire) and we describe the viral genome sequencing and analysis of the EHDV-8, BTV-8 and BTV-4 circulating strains.

## 2 Materials and methods

### 2.1 Preliminary epidemiological investigations

Because of the unexpected high number of clinical cases of BTV-8 in sheep and cattle and the introduction and quick spread of EHD cases in cattle, preliminary epidemiological investigations were conducted. In order to estimate farm-level of morbidity and mortality of BTV-8 and EHDV-8 infections, epidemiological surveys, based on phone call and epidemiological questionnaire, were organized and coordinated at the national level by the Groupement de Défense Sanitaire France (GDS France) and operated by local GDS branches.

BTV-8 survey was operated in October 2023 by local branch of GDS where BT cases were reported (GDS of Aveyron). Data were collected from 22 cattle farms and 22 sheep farms randomly selected among farms with at least one confirmed clinical case of BT before September 3, 2023 (diagnostic confirmation by rt-RT-PCR) and for which a suspicion form had been completed by the veterinarian. EHDV-8 survey was carried out by two local GDS branches in the Pyrenees, where EHD cases were reported (GDS of Pyrénées-Atlantiques and GDS of Hautes-Pyrénées). Data were collected between the end of October 2023 and the beginning of December 2023, from 74 cattle farms randomly selected among farms that had at least one confirmed EHD case before October 20, 2023. All data were then compiled and analyzed by GDS France and the French platform for epidemiological surveillance in animal health (ESA plateform).

### 2.2 Samples origin, molecular diagnosis and virus isolation

All EHDV-8, BTV-8 and BTV-4 infected blood samples collected on the field and viral isolates obtained from blood samples were analyzed in this study. Blood samples or spleens from animals located in mainland France were mainly collected from symptomatic or dead animals (sheep and cattle) during the BT and EHD outbreaks, but also from asymptomatic animals undergoing animal movement control testing. Regarding Corsica, blood samples from all suspected clinical cases of BT (or EHD) were analyzed at the National Reference Laboratory (NRL). In addition, samples were collected from asymptomatic cattle from random testing in slaughterhouses. Regarding wildlife, the NRL received 29 samples (28 spleens and 1 EDTA blood sample) from 29 wild cervids found dead in forests (or close to) EHDV-infected areas, between September and December 2023 via the SAGIR (“surveillance of wildlife in France”) network. All these samples were sent to the NRL for diagnostic using group-specific RT-qPCRs. An in house adapted version of the RT-qPCR targeting the S10 of the circulating BTV serotypes described in the chapter 3.1.3. of the terrestrial manual of the World Organisation for Animal Health was used (WOAH - World Organisation for Animal Health, 2024). The simplex assay was converted to a duplex RT-qPCR allowing both the detection of BTV sequences and the beta-actin RNA of ruminant cells (housekeeping gene) as internal control as previously described (Viarouge et al., 2015). A pan-EHD RT-qPCR targeting the S9 of known EHDV serotypes was used, as described above (Viarouge et al., 2015). RT-qPCR reactions were performed using the AgPath-ID™ One-Step RT-PCR mastermix (Thermofisher, USA) as manufacturer's instructions.

For selected RT-qPCR-positive samples, a two-step viral isolation was then carried out as previously described (Acevedo et al., 2024). First *Culicoides* cells (KC cells) were inoculated with BTV or EHDV infected blood samples, tested by RT-qPCR and when positive, the KC culture cell supernatants were inoculated on BSR cells until the appearance of cytopathic effects.

Blood samples with high viral loads (pan-BTV or –EHDV RT-qPCR Ct values under 25) or cell culture supernatants were selected for NGS analysis. Table 1 gives details of the samples selected. Except blood 11,400 (asymptomatic bovine), all selected samples came from animals with BT or EHD clinical signs.

**Table 1:** Sample description (type, host, date of sampling, location, genomic completion rate) and NCBI sequence accession number (AN).

Virus-serotype	Sample id.	Sample type	Host	Date of sampling	French department (code)	Genomic coverage (%)	AN
BTV-4	10,786	Isolate	Sheep	2023/10/06	Corsica	100.0	PP199191 - 200
BTV-4	11,114	Blood	Sheep	2023/10/17	Corsica	98.8	PP199201 - 10
BTV-4	11,115	Blood	Sheep	2023/10/17	Corsica	99.7	PP199211 - 20
BTV-4	13,004	Blood	Sheep	2023/11/14	Corsica	100.0	PP199221 - 30
BTV-4	13,006	Blood	Sheep	2023/11/14	Corsica	99.6	PP199231 - 40
BTV-4	13,408	Blood	Sheep	2023/11/20	Corsica	99.4	PP199241 - 50
BTV-8	8644	Isolate	Cattle	2023/08/22	Aveyron (12)	100.0	PP199251 - 60
BTV-8	8645	Isolate	Cattle	2023/08/22	Aveyron (12)	100.0	PP199261 - 70
BTV-8	8646	Isolate	Cattle	2023/08/22	Aveyron (12)	100.0	PP199271 - 80
BTV-8	8875	Blood	Sheep	2023/08/29	Aveyron (12)	100.0	PP199281 - 90
BTV-8	10,102	Blood	Sheep	2023/09/23	Corsica	100.0	PP199291 - 300
BTV-8	10,105	Blood	Sheep	2023/09/23	Corsica	100.0	PP199301 - 10
BTV-8	11,400	Blood	Cattle	2023/10/24	Mayenne (53)	99.6	PP199311 - 20
EHDV-8	9421	Isolate	Cattle	2023/09/07	Pyrénées-Atl. (64)	100.0	PP199321 - 30
EHDV-8	9982	Blood	Cattle	2023/09/16	Hautes-Pyrénées (65)	100.0	PP199331 - 40
EHDV-8	13,392	Blood	Cattle	2023/11/16	Loire-Atlantique (44)	100.0	PP199341 - 50

### 2.3 RNA extraction for NGS sequencing and control

According to the sample type, 140 µL of infected cell supernatants or 100µl of fresh blood were used for total RNA extraction using the QIAamp Viral RNA Mini Kit (Qiagen, Hilden, Germany), in accordance with to the manufacturer's instructions (without addition of RNA carrier). Extracted RNA was eluted in 60 µL of nuclease-free water and 5 µL of RNA (denatured 95 °C; 3 min) were controlled by RT-qPCR assays as described above.

### 2.4 Sample preparation using SISPA approach for whole genome sequencing

Whole genome sequencing of BTV and EHDV strains was performed using a Sequence-independent single-primer amplification (SISPA) approach, slightly modified to enrich orbiviruses sequences, and the Oxford Nanopore MinION technology (Acevedo et al., 2024; Chrzastek et al., 2017; Djikeng et al., 2008). Overrepresented rRNA from the host were depleted using the NEBNext® rRNA Depletion Kit with RNA Sample Purification Beads (New England Biolabs, USA). Retro-transcription, second strand synthesis and amplification were carried out according to the SISPA approach including the use a combination of random-tagged primers (FR26RV-N: GCCGGAGCTCTGCAGATATCNNNNNNN) (Djikeng et al., 2008), and specific-tagged primers (FR-BT_F: GCCGGAGCTCTGCAGATATCGTTAAAN and FR-BT_R: GCCGGAGCTCTGCAGATATCGTAAGTN) targeting the conserved extremities of the ten orbiviruses genomic segments (Peserico et al., 2019; Sghaier et al., 2022). Briefly, dsRNA samples were denatured (95 °C, 5 min) and set at 4 °C for 3 min. Then, RNAs were reverse transcribed into cDNA with Reverse Transcriptase SuperScript™ IV Kit (Life Technologies, USA). Second-strand synthesis (SSB) of the cDNA was performed by adding 1 µL (5 U) of polymerase, Klenow Fragment (3′→5′ exo-) (New England Biolabs, USA), at 37 °C, 60 min and 10 min at 75 °C. Tenfold diluted dsDNA was amplified using the FR20_Rv primer targeting the SISPA tag (FR20_Rv: GCCGGAGCTCTGCAGATATC) (Djikeng et al., 2008) and the Q5® Hot Start High-Fidelity DNA Polymerase (New England Biolabs, USA). Produced amplicons were purified using HighPrep PCR Clean-up System (MagBio Genomics Inc., USA) and eluted in 30 µL of DNase free water. Finally, total dsDNA was quantified with the dsDNA High Sensitivity (HS) assay Kit and Orbivirus dsDNA checked using BTV and EHDV specific RT-qPCRs (without RT step). The quality and average size of the amplicons were assessed using a TapeStation 2200 system (Agilent Technology, USA) and the Genomic DNA ScreenTape kit (Agilent Technologies, USA).

### 2.5 Nanopore sequencing and phylogenetic analysis

Sequencing libraries were prepared using the NBD114.24 Native Barcoding kit (Oxford Nanopore Technologies, UK) with native barcoding available on website (version “ligation-sequencing-amplicons-native-barcoding-v14-sqk-nbd114–24-NBA_9168_v114_revM_

15Sep2022-minion”). Beads ratio was kept to 1.8X along the library preparation steps. Fifty fmoles of final pooled library were loaded onto a FLO-MIN114 R10.4.1 flow cell. A 72h run was conducted with standard settings and the MinKNOW software (version 23.07.8). Raw reads were basecalled and demultiplexed using GUPPY (version 6.4.6) and the highly accurate model. Genomic sequences were produced using a custom mapping workflow on Geneious Prime (version 2022.0.2). First, SISPA labels were removed (20 bases, in 5′ and 3′) from the read sequences. Only 150 to 9000 bp reads were filtered and mapped using minimap2 (kmer length of 10) and mapped on the reference sequences OQ860834 – 43, OK018210 - 19, OP897265, OP897541 - 44, AM745072 and OP897546 - 49. Most of the consensus sequences were produced with a minimum of 100 of sequencing depth. Genome accession numbers are available in Table 1.

The identification of the closest nucleotide homology available in GenBank nt data-base was performed using the online BLAST search tool. Alignment and phylogenetic analyses were performed using MEGA X (version 10.2.0) (Kumar et al., 2018). Alignments were achieved using MUSCLE (Edgar, 2004), and phylogenetic trees reconstructed using the Maximum Likelihood method and Tamura–Nei model, with a bootstrap of 1000 (Tamura and Nei, 1993). The trees were drawn to scale, with branch lengths established by measuring in the number of substitutions per site. All positions containing gaps and missing data were eliminated.

### 2.6 RT-qPCR specific of the BTV-8 2023 strain

A duplex RT-qPCR assay specifically targeting a region of the BTV segment 2 and the beta-actin RNA (internal control as described above), was implemented at the Friedrich-Loeffler-Institut to differentiate the newly introduced and the endemic BTV-8 strains. The nucleotide sequences for the forward and reverse primers of the target are, respectively, BTV8-FRA23–1982F: ATT GAT CCC AAC ATT GAT ATT GAG and BTV8-FRA23–2071R: CGA TTT TCA AAC AAA TAG TCA AAT ACA; and for the probe: FAM-ATA probe ATC GAT GTC TCG CAA CTG AT-BHQ1. RT-qPCR reactions were performed using the AgPath-ID™ One-Step RT-PCR mastermix (Thermofisher, USA), following the manufacturer's recommendations for primers and probe concentrations and the following thermal program: 10 min, 45 °C; 10 min, 95 °C and 42 PCR cycles (15 sec, 95 °C; 20 sec, 58 °C and 30 sec, 72 °C).

## 3 Results

### 3.1 BT and EHD outbreaks in France, season 2023

#### 3.1.1 BT outbreaks in France, season 2023

Since the beginning of August 2023 (week 32), several clinical cases of BT (BTV-8) were first detected in cattle and sheep in the southern part of Massif Central, Aveyron department. Animals showed hyperthermia, anorexia, locomotion difficulties, members oedema, scabs on the muzzle, ulcerations in the mouth, and on the udder, hypersalivation, weight loss, conjunctivitis, nasal discharge and a blue tongue (sheep). Within weeks, the virus spread rapidly to surrounding flocks and departments. The GDS survey in Aveyron showed, one to two months after herds infection, high inter-herd variability in adult cattle morbidity, ranging from 1 % to around 73 %, and from 0.3 % to 47 % in adult sheep. Inter-herd variability in mortality ranged from 0 % to 5 % in adult cattle and from 0 % to 31 % in adult sheep. Almost all affected sheep farms (21/22) suffered animal losses. Finally, in September (week 39), following suggestive BT clinical signs reports, BTV-8 was detected in sick sheep from the south of the Corsican Island. On the following day (week 41), others sick sheep were found to be positive for BTV-4. By the end of December 2023, among BT outbreaks in Corsica, a total of 25 sheep flocks, one cattle and one goat were confirmed infected by BTV-8, nine sheep flock infected by BTV-4 and four sheep flocks one cattle co-infected BTV-8 and -4.

In order to determine whether the BTV-8 strain present in France since 2015 was still circulating in 2023 at the same time as the new BTV-8 2023 strain, a strain-specific screening was performed among a subset of BTV-8 infected blood samples received at the NRL for various reasons (clinical suspicion, export, confirmation of results from departmental laboratories, etc....). Two hundred and two BTV-8 infected animals (with symptoms or not) from 158 herds in mainland France were then tested with the RT-qPCR developed by the FLI, which specifically detect the BTV-8 2023 strains. Twenty nine of 202 animals were found infected with the endemic BTV-8 strain and 173/202 with the new BTV-8 strain. In Corsica, all the 89 BTV-8 infected animals tested were infected with the new BTV-8.

#### 3.1.2 EHD outbreaks in France, season 2023

In parallel, at the beginning of September 2023 (week 36), from two French departments of the Pyrenees, on the south west side bordering Spain, three bovines with clinical signs suggestive of BT, were found to be BTV negative by RT-qPCR but EHDV RT-qPCR positive. An EHDV-8 was identified and the virus spread rapidly to surrounding herds, with at least one-third of livestocks from the two infected departments found EHDV RT-qPCR-positive 1.5 months after its introduction and first detection. The clinical signs of EHD reported were similar to those described in Tunisia, Italy and Spain, including hyperthermia, anorexia, abatement, lameness, congestion, ulcers in the mouth, conjunctivitis, nasal discharge and lingual prolapse (Jiménez-Cabello et al., 2023; Ruiz-Fons et al., 2024). No EHD clinical signs were reported in sheep. Data collected from the field showed highly variable morbidity rate among adult cattle, ranging from 1.8 % to around 100 % depending on the herd size (until 40 animals per farm). More than a third of farms surveyed had more than 20 % of adult animals affected. Severe symptoms were also observed in two-years-old bovine and over. Negligible bovine mortality was observed on a collective scale, although some livestock suffered losses of several animals.

**Fig. 1:**
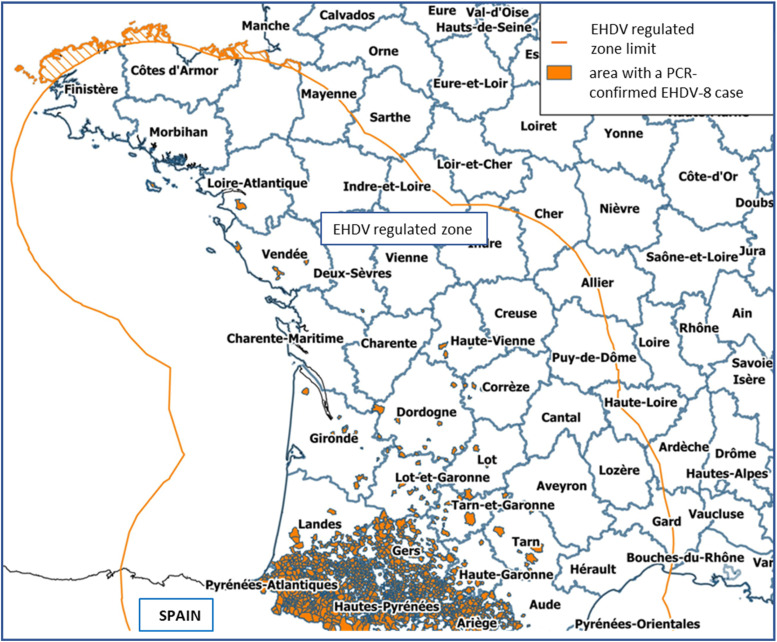
Map of the French municipalities infected by EHDV-8 in 2023, on the 31st of May, 2024. Source: Direction Générale de l'Alimentation / Ministère de l'Agriculture et de la Souveraineté Alimentaire.

To the date of May 31, 2024, 4310 outbreaks were reported in 20 French departments (Fig. 1). The vast majority of the EHD cases was located in the Pyrenees, while few outbreaks have been observed further north, along the Atlantic coast. EHDV-susceptible animals located within 150 km of a PCR-confirmed EHDV case were subject to PCR testing and/or quarantine before moving out of the EHDV-regulated zone.

### 3.2 Virus isolation and whole genome recovery

After passage through embryonated eggs or KC cells, isolates of BTV-4, 8 and EHDV-8 were obtained from the first samples received in the laboratory for clinical suspicions. At least one of these isolates (per serotype) was used for whole genome sequencing (Table 1). A total of 11 viremic blood samples were also selected at different times during the epizootic and in different departments (Table 1).

All these samples was sequenced using the SISPA – MinION sequencing approach. Complete genomes were obtained from the 5 isolates and 6 of the 11 blood samples sequenced. Percentage coverage for the other 5 bloods ranged from 98.8 to 99.7 %.

#### 3.2.1 Genomic sequences analysis of the BTV-8 strains

**Fig. 2:**
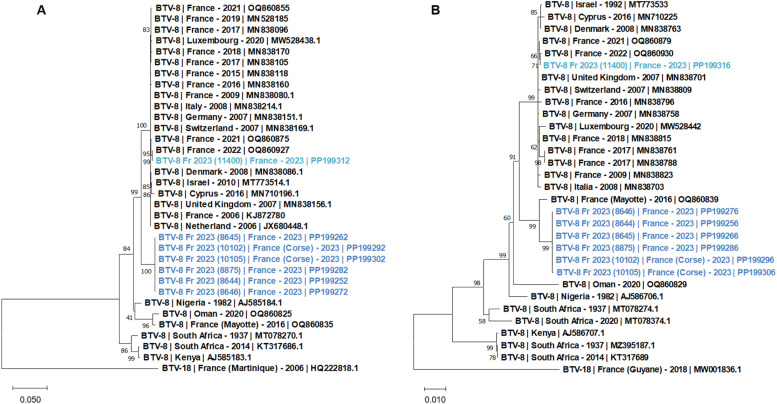
Phylogenetic analysis of S2 (A) and S6 (B) sequences of the French BTV-8 strains. Phylogenetic analysis of S2 (A) and S6 (B) sequences of BTV-8 strains using the maximum likelihood method and Tamura-Nei model with 1000 bootstrap replicates in MEGA X. This analysis involved 34 (A) and 31 (B) nucleotide sequences, there were a total of 2940 (A) and 1610 (B) positions in the final dataset. Bootstrap values appeared at the corresponding nodes. In the phylogenetic tree, GenBank sequences, BT serotype, country and year of sample collection are given. In dark blue, sequences from the newly introduced BTV-8 strain; in light blue sequences from the endemic BTV-8 strain detected in 2023.

The sequence and phylogenetic analysis of the BTV-8 genomes recovered in this study revealed the circulation of two distinct BTV-8 strains. A newly introduced BTV-8 strain, with the six genomes obtained from the samples collected in Aveyron and Corsica where severe BT cases were reported, and the endemic BTV-8 strain, with the genome recovered from an asymptomatic cattle blood sample. The new BTV-8 strain sequences, referred as the “2023-outbreak” BTV-8 group in Table 2, displayed very high nucleotide identity (NI), with rate ranging from 99.8 to 100 % (Table 2) and almost consistently 100 % of amino acid identity (AAI) (except 99.8 % of AAI for NS1 sequences) (data not shown). The newly introduced BTV-8 strain only shared 93.5 % (S8) to 97.2 % (S1) of NI with the endemic BTV-8 strain sequences (Table 2). Yet, most of the AA sequences were conserved between the two group (97.4 to 100 %) except for VP6 (93.9 %) and NS5 (91.5 %). Almost all the genomic sequences of the new BTV-8 strain displayed closest relationship with strains identified in Middle East, Mayotte or South Africa (Fig. 2). As expected, the closest homologies identified for the endemic strain corresponded to the BTV-8 strains previously reported in France and other European countries.

**Table 2:** First closest homology of the BTV-8 genomic sequences found in GenBank with 99–100 % of sequence coverage (S: segment; nt: nucleotide AA: Amino acid; Id %: Percentage identity; AN: accession number; Id %: percentage of NI).

S	BTV-8 Strain group	nt (AA) Id % between groups	Subject AN	Subject	nt Id ( %)
S1	“2023-outbreak” BTV-8 group	97.2 (100.0)	MN837926	BTV-8 GER2006-BH438–23	97.6 - 97.7
	“Endemic” BTV-8 group		OQ860926	BTV-8 2022 (12,718)	99.9
S2	“2023-outbreak” BTV-8 group	96.4 (98.6)	KJ872780	BTV-8 Ardennes 2006	96.8
	“Endemic” BTV-8 group		OQ860927	BTV-8 2022 (12,718)	99.9
S3	“2023-outbreak” BTV-8 group	94.2 (99.6)	OR603994	BTV-3/NET2023	98.6 - 98.7
	“Endemic” BTV-8 group		OQ860928	BTV-8 2022 (12,718)	99.9
S4	“2023-outbreak” BTV-8 group	95.2 (98.4)	KP821279	BTV-2 TUN2000/01	98.5 - 98.6
	“Endemic” BTV-8 group		OQ860929	BTV-8 2022 (12,718)	100
S5	“2023-outbreak” BTV-8 group	93.8 (99.6)	KP821370	BTV-1 LIB2007/06	98 - 98.1
	“Endemic” BTV-8 group		OQ860925	BTV-8 2022 (12,718)	99.8
S6	“2023-outbreak” BTV-8 group	97.6 (99.8)	OQ860839	BTV-8 2016 (5191)	98.9 - 99
	“Endemic” BTV-8 group		OQ860899	BTV-8 2022 (12,717)	100
S7	“2023-outbreak” BTV-8 group	96.5 (100.0)	MG255595	BTV-24 /O.aries-tc/ZAF/VR25_2017	98.4 - 98.5
	“Endemic” BTV-8 group		OQ860850	BTV-8 2021 (9957)	99.8
S8	“2023-outbreak” BTV-8 group	93.5 (97.7)	MG255457	BTV-5/O.aries-tc/ZAF/2011	98.6
	“Endemic” BTV-8 group		OQ860901	BTV-8 2022 (12,717)	100
S9	“2023-outbreak” BTV-8 group	95.1 (VP6 : 93.9 / NS4 : 97.4)	OK507983	BTV-9 ISR-1763/3/19	97.6 - 97.7
	“Endemic” BTV-8 group		OQ860933	BTV-8 2022 (12,718)	100
S10	“2023-outbreak” BTV-8 group	96.0 (NS3 : 100.0 / NS5 : 91.5)	MT043227	BTV-13/O.aries-tc/ZAF/2017	97.7 - 97.8
	“Endemic” BTV-8 group		OQ860934	BTV-8 2022 (12,718)	99.8

The phylogenetic relationships of S2 (VP2) and S6 (VP5) of the BTV-8 strains described in this study confirmed the clustering of the sequences accordingly to their epidemiological context. The sequences corresponding to the newly introduced BTV-8 strain clearly formed a different cluster from the one including the endemic BTV-8 strain sequences detected in France and other European countries (Fig. 2).

#### 3.2.2 Genomic sequences analysis of the BTV-4 strain from Corsica

All the BTV-4 genomes recovered from the six samples collected in Corsica from BT outbreaks allowed the identification of one BTV-4 strain. Sequences were highly conserved with NI ranging from 99.7 to 100 % and more than 99.9 % of AAI (Table 3). The closest homologies identified, with high level of nucleotide conservation, were genomic sequences from BTV-4 identified in Kosovo and Macedonia and previous BTV-4 identified in Corsica in 2017 (S10).

**Table 3:** First closest homology of the BTV-4 genome sequences found in GenBank with 99–100 % of sequence coverage (S: segment; nt: nucleotide AA: Amino acid; Id %: Percentage identity; AN: accession number; Id %: percentage of NI).

S	nt (AA) Id % between BTV-4 strains	Subject AN	Subject	Id ( %)
S1	99.9 - 100 (100)	MT879201	BTV-4 MKD2020/06	99.8
S2	99.9 - 100 (100)	OP186407	BTV-4 KOS2020/02	99.7 - 99.8
S3	100 (100)	OP186408	BTV-4 KOS2020/02	99.9 - 100
S4	99.9 - 100 (100)	OP186419	BTV-4 KOS2014/01	99.7 - 99.8
S5	99.9 - 100 (100*)	OP186410	BTV-4 KOS2020/02	99.6 - 99.7
S6	99.9 - 100 (99.8–100)	OP186411	BTV-4 KOS2020/02	99.3 - 99.4
S7	99.9 - 100 (100)	OP186422	BTV-4 KOS2014/01	99.7
S8	100 (100.0)	OP186413	BTV-4 KOS2020/02	99.5 - 99.6
S9	99.8 - 100 (VP6: 99.4–100 / NS4: 98.7–100)	OP186414	BTV-4 KOS2020/02	99.5 - 99.8
S10	99.7 - 100 (NS3: 100 / NS5: 100)	MG944826	BTV-4/17–15 (8287)	99.9 - 100

**Fig. 3:**
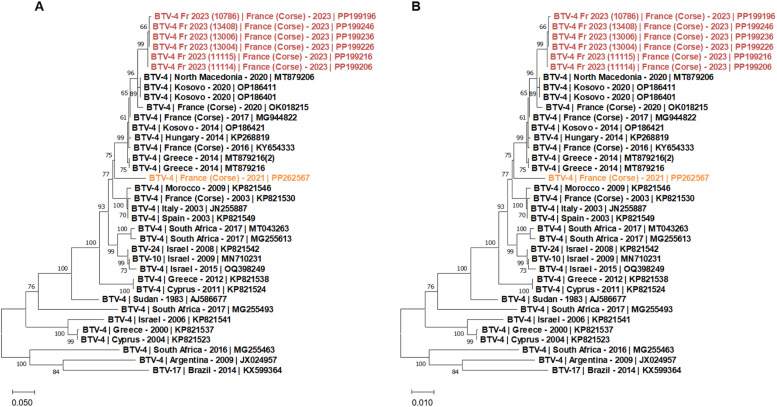
Phylogenetic analysis of S2 (A) and S6 (B) sequences of BTV-4 strains. Phylogenetic analysis of S2 (A) and S6 (B) sequences of the BTV-4 strains using the maximum likelihood method and Tamura-Nei model with 1000 bootstrap replicates in MEGA X. This analysis involved 32 (A) and 36 (B) nucleotide sequences, there were a total of 2903 (A) and 1609 (B) positions in the final dataset. Bootstrap values appeared at the corresponding nodes. In the phylogenetic tree, GenBank sequences, BT serotype, country and year of sample collection are given. In red, sequences from the BTV-4 strain identified in Corsica in 2023 and described in this study; in orange, sequences of the BTV-4 strain detected in 2021 in Corsica.

The phylogenetic relationship of S2 (VP2) and S6 (VP5) of the BTV-4 described in this study with other BTV-4 sequences published from France, Africa, Middle East and Argentina confirmed these observations (Fig. 3). The BTV-4 sequences found in 2023 clustered with sequences from BTV-4 from the Balkans region, including sequences from Greece, Kosovo, Macedonia between at least 2014 and 2020 and the previous BTV-4 strain identified in Corsica between 2016 and 2020. This clustering was observed among all the genomic segments and strongly suggest the circulation of the same BTV-4 strain in this geographical area (supplementary data). Interestingly, the BTV-4 strain identified in Corsica and responsible of the latest BT cases reported on the island in 2021 clearly formed a distinct cluster.

#### 3.2.3 Genomic sequences of the EHDV-8 strain introduced in mainland france

**Fig. 4:**
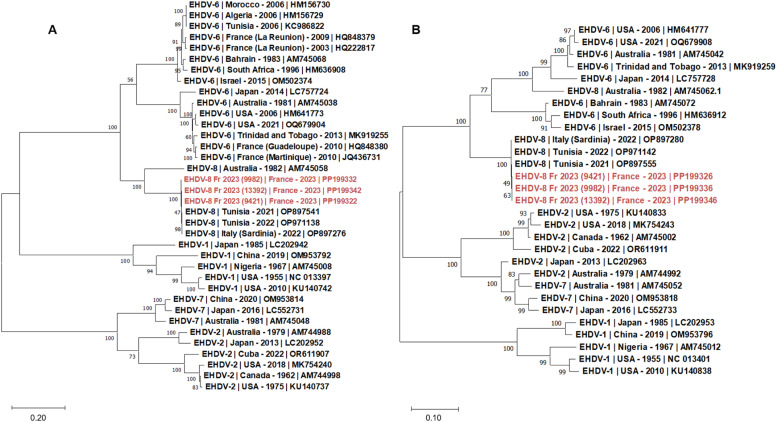
Phylogenetic analysis of S2 (A) and S6 (B) sequences of the EHDV strains. Phylogenetic analysis of S2 (A) and S6 (B) sequences of EHDV strains using the maximum likelihood method and Tamura-Nei model with 1000 bootstrap replicates in MEGA X. This analysis involved 36 (A) and 29 (B) nucleotide sequences, there were a total of 3040 (A) and 1585 (B) positions in the final dataset. Bootstrap values appeared at the corresponding nodes. In the phylogenetic tree, GenBank sequences, EHD serotype, country and year of sample collection are given. In red, sequences from the EHDV-8 strain introduced in France in 2023.

Three EHDV-8 infected blood samples were analyzed, two (sampled week 36 and 37) from the south west of France (area of introduction of the virus) and one from the northern area (Loire-Atlantique department, sampled week 45). As expected, the analysis of the three EHDV genomes recovered in this study revealed the introduction in France of the same EHDV-8 strain that was firstly reported in Tunisia in 2021 and which has spread to the continent through Italy and Spain (Table 4). These three genomic sequences displayed high level of conservation with NI, with identity ranging from 99.7 to 100 % for nt and more than 99.7 % of for AA and clustered together (Table 4, Fig. 4).

**Table 4:** First closest homology of the EHDV-8 genome sequences found in GenBank with 99–100 % of sequence coverage (S: segment; nt: nucleotide AA: Amino acid; Id %: Percentage identity; AN: accession number; Id %: percentage of NI).

S	nt (AA) Id % between EHDV-8 strains	Subject AN	Subject	Id (%)
S1	99.9 (99.7–99.9)	OP897550	EHDV-8/Deer TUN2021	99.9
S2	99.8 - 100 (99.8 - 100)	OP897541	EHDV-8/60 TUN2021	99.7 - 99.8
S3	99.9 - 100 (99.9 - 100)	OP897552	EHDV-8/Deer TUN2021	99.8 - 99.9
S4	99.7 - 99.8 (99.7 - 99.8)	OP897553	EHDV-8/Deer TUN2021	99.7 - 99.8
S5	99.9 (99.8 - 100)	OP971131	EHDV-8/Cattle Bizerte TUN2022	99.9 - 100
S6	100 (100)	OP897555	EHDV-8/Deer TUN2021	99.9
S7	99.9 - 100 (100)	OP897556	EHDV-8/Deer TUN2021	99.9 - 100
S8	100 (100)	OP971144	EHDV-8/Cattle Tunis TUN2022	99.9
S9	100 (VP6: 100 / NS4: 100)	OP897558	EHDV-8/Deer TUN2021	100
S10	100 (NS3: 100)	OP971146	. EHDV-8/Cattle Tunis TUN2022	99.9

Interestingly, S2 sequences of the Mediterranean EHDV-8 strains only shared around 77 % of NI with the Australian strain (data not shown). The analysis of the phylogenetic relationships of the other segments (S1, S3-S5, S7-S10) showed that the sequences of the Mediterranean EHDV-8 strain clustered together with different EHDV serotypes (1, 4, 6 and 7) reported in African countries including Israel, Bahrain, South Africa, and Nigeria (supplementary data).

## 4 Discussion

In this study, we presented the exceptional nature of EHD and BT epizootics in mainland France and Corsica (only for BT) in 2023 and characterized the genomic sequences of the circulating EHDV and BTV strains.

The sequencing approach used in this study, the combination of SISPA and Oxford Nanopore sequencing, enabled rapid identification of circulating genotypes. Compared to a previous study focusing on viral isolates, we demonstrated here the ability of the method to recover complete genome directly from blood samples with a high to mid EHDV or BTV viral load (most of the Pan RT-qPCR Ct values were between 18 and 22, up to 25) (Acevedo et al., 2024). Sequencing genomes directly from field samples allowed to by-pass the time and resource consuming viral isolation steps which is a real asset in an epizootic context.

Two distinct BTV-8 strains were identified by RT-qPCR and genome sequencing in mainland France, including the enzootic strain identified since 2015 and a new BTV-8 strain of unknown origin. Interestingly, all genomics sequences obtained from samples collected on symptomatic animals located within the outbreaks area belonged to the new BTV-8 strain. Only the new BTV-8 strain was detected in Corsica. Whether the emergence of the new BTV-8 strain in both mainland France (south part of Massif Central) and Corsica resulted from two independent events is unclear. But, animal movements from the epicenter area of the BT outbreaks in mainland France, to Corsica before the detection of the new BTV-8 strains on the island were reported, suggesting that the continent was first infected. The origin of the new BTV-8 strain responsible of BT outbreaks in 2023 remains to be investigated. Phylogenetic analysis of the genomic sequences, together with high homologies with sequences predominantly detected in the Mediterranean basin and South Africa, strongly suggest an African origin. Interestingly, S3 of the new BTV-8 strain described in France displayed the strongest homology with S3 of the BTV-3 strain that emerged in the Netherlands, almost concomitantly. The ten segments of this BTV-3 strain also show the strongest homologies with strains from South Africa and the Mediterranean basin. Could the emergence in Europe of the virulent BTV-3 and BTV-8 strains, with similar African origins and within a few weeks of each other, be linked to a same phenomenon? Unfortunately, the lack of genomic sequences available on database, especially when considering the expected large diversity of BTV strains circulating in Africa, limits greatly the phylodynamic analysis of BTV.

In Corsica, we also reported the circulation in 2023 of the BTV-4 strain previously detected on the island in 2016, 2017 and 2020. This BTV-4 strain was also identified in different countries from the Balkan area for several years (Sailleau et al., 2018). Whether the reemergence of this strain in Corsica in 2023 came from a new introduction from the continent or a silent circulation on the island remains unclear.

Co-circulation of BTV strains of different origins, with diverse genotypes, raise the risk of reassortment. On mainland France, the two different BTV-8 strains already present overlapping area of circulation, thus the stability of the BTV 8 strain circulating on the territory since 2015 might be challenged in the future. Similar concerns should be raised in Corsica where two different BTV serotypes co-circulate and where co-infected BTV-4 and 8 animals were already identified. For the time being, we have not observed any reassortments between the different BTV strains present in 2023 in mainland France and Corsica, based on random sampling of fully sequenced strains. These potential reassortment events and their impact on strain fitness and phenotype will need to be investigated during the probable re-emergence and spread of these different strains in the coming years.

Finally, we reported the first introduction of EHDV, serotype 8, in France. EHD cases were reported from two departments constituting the French side of the Pyrenees, strongly supporting an introduction from Spain, where the virus circulates since 2022 (Ruiz-Fons et al., 2024). Genomes of EHDV-8 reported in Tunisia, Italy, and now France were highly conserved as expected (Jiménez-Cabello et al., 2023; Lorusso et al., 2023; Sghaier et al., 2022). According to S2 and S6 sequences, this EHDV-8 strain displayed closest relationship with the only other EHDV-8 reported in 1982 in Australia. When analyzing the phylogeny of the others genomics segments, sequences of the EHDV-8 strain circulating in Tunisia and Europe clustered with sequences from different EHDV serotypes circulating in Africa since a long time (supplementary data). As already reported for other strains, reassortment of local EHDV strains might be the mechanism behind the emergency of this particular EHDV-8 genotype (Allison et al., 2012; Rajko-Nenow et al., 2019). Yet, the reason why this EHDV-8 strain is responsible for outbreaks in cattle since 2021, and the factors which allowed its spread towards Europe, contrarily to the other North African EHDV strains, remains to be investigated.

The GDS surveys included data collected one to two months after the EHDV-8 and BTV-8 emergence on mainland France and aimed to provide rapidly after the beginning of the emergence some epidemiological insights on the health impact of these new orbiviruses strains. Few EHD or BT cases were reported on young animals (under 6 months old). The most severe clinical signs and mortalities were more clearly and more frequently observed in adults, in sheep infected with BTV-8 and in cattle infected with EHDV-8 or BTV-8. Although morbidity and mortality might be underestimated as the survey was deployed before the end of the season, the remarkable rates reported by the GDS surveys recall the epizootic caused by the BTV-8 strain in 2006, a strain that is still circulating in 2023 with rare associated symptoms for several years. The role of small domestic ruminants in the epidemiology of EHDV appears to be negligible. As shown by the experimental study carried out with the EHDV-8 strain, EHDV viremia in sheep appears to be very short, with no clinical signs observed in infected animals (Spedicato et al., 2023). During the EHD epizootic in France in 2023, no clinical suspicion was reported in goats and the few clinical suspicions reported in sheep were not confirmed by EHDV specific RT-qPCR. Few data are available on EHDV circulation in the wildlife. Four spleens, collected from dead Pyrenean chamois (*Rupicapra pyrenaica*), roe or red deer, were found positive when tested by EHDV specific RT-qPCR, with high viral loads, suggesting recent EHDV infection in these animals. In Spain, a recent study reported severe and fatal EHD cases in European red deer (*Cervus elaphus*) and a seropositivity rate of 6 % in a cross-sectional serological study conducted on 592 cervids (Ruiz-Fons et al., 2024). All together, these data suggest that the impact of EHD in wildlife (in Spain and in the South West of France) seems low but, in accordance with the conclusions of the Spanish study, this impact on wildlife can be variable depending on many parameters (species, *Culicoides* population, eco-environment, contact with cattle, wildlife ruminant species and their density, etc.) and deserve further investigation.

To conclude, the exceptional character of the epizootics, regarding the diversity of the strains involved, the introduction of EHDV together with the apparition of a new BTV-8 strain, stressed out the need for real time epidemiological investigations. The origin of these viruses, the factors involved in their emergency and spread are too long-unanswered questions.

## Author statement

No AI technologies (large language models or otherwise) were used in the analysis or preparation of this manuscript.

## Funding

This research was funded by the PREPMEDVET project (grant number ANR-20-SEBM-0004) and by the European Partnership on Animal Health and Welfare (the Union's Horizon Europe Project 101136346 EUPAHW).

## CRediT authorship contribution statement

**Mathilde Gondard:** Writing – review & editing, Writing – original draft, Methodology, Investigation, Formal analysis, Data curation, Conceptualization. **Lydie Postic:** Writing – review & editing, Investigation. **Emmanuel Garin:** Writing – review & editing, Resources, Investigation. **Mathilde Turpaud:** Writing – review & editing, Investigation. **Fabien Vorimore:** Resources, Methodology. **David Ngwa-Mbot:** Writing – review & editing, Resources, Investigation. **Mai-Lan Tran:** Resources, Methodology. **Bernd Hoffmann:** Writing – review & editing, Resources. **Charlotte Warembourg:** Writing – review & editing, Resources, Investigation. **Giovanni Savini:** Writing – review & editing, Resources. **Alessio Lorusso:** Writing – review & editing, Resources. **Maurilia Marcacci:** Writing – review & editing, Resources. **Arnaud Felten:** Resources, Investigation. **Aurélie Le Roux:** Investigation. **Yannick Blanchard:** Writing – review & editing, Investigation. **Stephan Zientara:** Writing – review & editing. **Damien Vitour:** Writing – review & editing. **Corinne Sailleau:** Writing – review & editing, Visualization, Supervision, Project administration, Conceptualization. **Emmanuel Bréard:** Writing – review & editing, Writing – original draft, Visualization, Supervision, Project administration, Funding acquisition, Conceptualization.

## Declaration of competing interest

The authors declare that they have no known competing financial interests or personal relationships that could have appeared to influence the work reported in this paper.

## Data Availability

All data used is publicly available on GenBank or in the published literature.

## Appendix Supplementary materials

Image, application 1

Image, application 2
