# Supplementary material for: Exceptional Bluetongue virus (BTV) and Epizootic hemorrhagic disease virus (EHDV) circulation in France in 2023
Source: Virus Res. 2024 Nov 1;350:199489. doi: 10.1016/j.virusres.2024.199489 (PMC11565556; doi:10.1016/j.virusres.2024.199489)
Supplement: Supplementary file 1 [file mmc1.docx]

**Supplementary data 1. Phylogenetic analysis of the BTV-8 and BTV-4 strains, segments 1, 3, 4, 5, 7, 8, 9 and 10.**

**Figure S1: Phylogenetic analysis of S1 sequences of the BTV strains analyzed in this study.** Phylogenetic analysis of S1 sequences of BTV using the Maximum Likelihood method and Tamura-Nei model (1000 replicates). This analysis involved 82 nucleotide sequences; there were a total of 3867 positions in the final dataset. In the phylogenetic tree, GenBank sequences, species designations and strain names are given. Bootstrap values appeared at the corresponding nodes. In the phylogenetic tree, Genbank sequences, bluetongue serotype, country and year of sample collection are given. In dark blue and light blue, sequences from the new BTV-8 strain and the endemic BTV-8 strain respectively; in red and orange, sequences from the BTV-4 strain described in Corsica in 2023 and 2021 respectively.

**Figure S2: Phylogenetic analysis of S3 sequences of the BTV strains analyzed in this study.** Phylogenetic analysis of S3 sequences of BTV using the Maximum Likelihood method and Tamura-Nei model (1000 replicates). This analysis involved 82 nucleotide sequences; there were a total of 2719 positions in the final dataset. In the phylogenetic tree, GenBank sequences, species designations and strain names are given. Bootstrap values appeared at the corresponding nodes. In the phylogenetic tree, Genbank sequences, bluetongue serotype, country and year of sample collection are given. In dark blue and light blue, sequences from the new BTV-8 strain and the endemic BTV-8 strain respectively; in red and orange, sequences from the BTV-4 strain described in Corsica in 2023 and 2021 respectively.

**Figure S3: Phylogenetic analysis of S4 sequences of the BTV strains analyzed in this study.** Phylogenetic analysis of S4 sequences of BTV using the Maximum Likelihood method and Tamura-Nei model (1000 replicates). This analysis involved 82 nucleotide sequences; there were a total of 1755 positions in the final dataset. In the phylogenetic tree, GenBank sequences, species designations and strain names are given. Bootstrap values appeared at the corresponding nodes. In the phylogenetic tree, Genbank sequences, bluetongue serotype, country and year of sample collection are given. In dark blue and light blue, sequences from the new BTV-8 strain and the endemic BTV-8 strain respectively; in red and orange, sequences from the BTV-4 strain described in Corsica in 2023 and 2021 respectively.

**Figure S4: Phylogenetic analysis of S5 sequences of the BTV strains analyzed in this study.** Phylogenetic analysis of S5 sequences of BTV using the Maximum Likelihood method and Tamura-Nei model (1000 replicates). This analysis involved 82 nucleotide sequences; there were a total of 1608 positions in the final dataset. In the phylogenetic tree, GenBank sequences, species designations and strain names are given. Bootstrap values appeared at the corresponding nodes. In the phylogenetic tree, Genbank sequences, bluetongue serotype, country and year of sample collection are given. In dark blue and light blue, sequences from the new BTV-8 strain and the endemic BTV-8 strain respectively; in red and orange, sequences from the BTV-4 strain described in Corsica in 2023 and 2021 respectively.

**Figure S5: Phylogenetic analysis of S7 sequences of the BTV strains analyzed in this study.** Phylogenetic analysis of S7 sequences of BTV using the Maximum Likelihood method and Tamura-Nei model (1000 replicates). This analysis involved 82 nucleotide sequences; there were a total of 795 positions in the final dataset. In the phylogenetic tree, GenBank sequences, species designations and strain names are given. Bootstrap values appeared at the corresponding nodes. In the phylogenetic tree, Genbank sequences, bluetongue serotype, country and year of sample collection are given. In dark blue and light blue, sequences from the new BTV-8 strain and the endemic BTV-8 strain respectively; in red and orange, sequences from the BTV-4 strain described in Corsica in 2023 and 2021 respectively.

**Figure S6: Phylogenetic analysis of S8 sequences of the BTV strains analyzed in this study.** Phylogenetic analysis of S8 sequences of BTV using the Maximum Likelihood method and Tamura-Nei model (1000 replicates). This analysis involved 82 nucleotide sequences; there were a total of 962 positions in the final dataset. In the phylogenetic tree, GenBank sequences, species designations and strain names are given. Bootstrap values appeared at the corresponding nodes. In the phylogenetic tree, Genbank sequences, bluetongue serotype, country and year of sample collection are given. In dark blue and light blue, sequences from the new BTV-8 strain and the endemic BTV-8 strain respectively; in red and orange, sequences from the BTV-4 strain described in Corsica in 2023 and 2021 respectively.

**Figure S7: Phylogenetic analysis of S9 sequences of the BTV strains analyzed in this study.** Phylogenetic analysis of S9 sequences of BTV using the Maximum Likelihood method and Tamura-Nei model (1000 replicates). This analysis involved 67 nucleotide sequences; there were a total of 731 positions in the final dataset. In the phylogenetic tree, GenBank sequences, species designations and strain names are given. Bootstrap values appeared at the corresponding nodes. In the phylogenetic tree, Genbank sequences, bluetongue serotype, country and year of sample collection are given. In dark blue and light blue, sequences from the new BTV-8 strain and the endemic BTV-8 strain respectively; in red and orange, sequences from the BTV-4 strain described in Corsica in 2023 and 2021 respectively.

**Figure S8: Phylogenetic analysis of S10 sequences of the BTV strains analyzed in this study.** Phylogenetic analysis of S10 sequences of BTV using the Maximum Likelihood method and Tamura-Nei model (1000 replicates). This analysis involved 82 nucleotide sequences; there were a total of 442 positions in the final dataset. In the phylogenetic tree, GenBank sequences, species designations and strain names are given. Bootstrap values appeared at the corresponding nodes. In the phylogenetic tree, Genbank sequences, bluetongue serotype, country and year of sample collection are given. In dark blue and light blue, sequences from the new BTV-8 strain and the endemic BTV-8 strain respectively; in red and orange, sequences from the BTV-4 strain described in Corsica in 2023 and 2021 respectively.
