# Supplementary material for: Exceptional Bluetongue virus (BTV) and Epizootic hemorrhagic disease virus (EHDV) circulation in France in 2023
Source: Virus Res. 2024 Nov 1;350:199489. doi: 10.1016/j.virusres.2024.199489 (PMC11565556; doi:10.1016/j.virusres.2024.199489)
Supplement: Supplementary file 2 [file mmc2.docx]

**Supplementary data 2. Phylogenetic analysis of the EHDV-8 strains, segments 1, 3, 4, 5, 7, 8, 9 and 10.**

**Figure S1: Phylogenetic analysis of S1 sequences of the EHDV-8 strain analyzed in this study.** Phylogenetic analysis of S1 sequences of EHDV-8 strains using the Maximum Likelihood method and Tamura-Nei model (1000 replicates). This analysis involved 34 nucleotide sequences; there were a total of 3907 positions in the final dataset. In the phylogenetic tree, GenBank sequences, species designations and strain names are given. Bootstrap values appeared at the corresponding nodes. In the phylogenetic tree, Genbank sequences, bluetongue serotype, country and year of sample collection are given. The french sequences investigated in the present study are marked in red.

**Figure S2: Phylogenetic analysis of S3 sequences of the EHDV-8 strain analyzed in this study.** Phylogenetic analysis of S3 sequences of EHDV-8 strains using the Maximum Likelihood method and Tamura-Nei model (1000 replicates). This analysis involved 33 nucleotide sequences; there were a total of 2700 positions in the final dataset. In the phylogenetic tree, GenBank sequences, species designations and strain names are given. Bootstrap values appeared at the corresponding nodes. In the phylogenetic tree, Genbank sequences, bluetongue serotype, country and year of sample collection are given. The french sequences investigated in the present study are marked in red.

**Figure S3: Phylogenetic analysis of S4 sequences of the EHDV-8 strain analyzed in this study.** Phylogenetic analysis of S4 sequences of EHDV-8 strains using the Maximum Likelihood method and Tamura-Nei model (1000 replicates). This analysis involved 34 nucleotide sequences; there were a total of 1935 positions in the final dataset. In the phylogenetic tree, GenBank sequences, species designations and strain names are given. Bootstrap values appeared at the corresponding nodes. In the phylogenetic tree, Genbank sequences, bluetongue serotype, country and year of sample collection are given. The french sequences investigated in the present study are marked in red.

**Figure S4: Phylogenetic analysis of S5 sequences of the EHDV-8 strain analyzed in this study.** Phylogenetic analysis of S5 sequences of EHDV-8 strains using the Maximum Likelihood method and Tamura-Nei model (1000 replicates). This analysis involved 34 nucleotide sequences; there were a total of 1656 positions in the final dataset. In the phylogenetic tree, GenBank sequences, species designations and strain names are given. Bootstrap values appeared at the corresponding nodes. In the phylogenetic tree, Genbank sequences, bluetongue serotype, country and year of sample collection are given. The french sequences investigated in the present study are marked in red.

**Figure S5: Phylogenetic analysis of S7 sequences of the EHDV-8 strain analyzed in this study.** Phylogenetic analysis of S7 sequences of EHDV-8 strains using the Maximum Likelihood method and Tamura-Nei model (1000 replicates). This analysis involved 34 nucleotide sequences; there were a total of 1039 positions in the final dataset. In the phylogenetic tree, GenBank sequences, species designations and strain names are given. Bootstrap values appeared at the corresponding nodes. In the phylogenetic tree, Genbank sequences, bluetongue serotype, country and year of sample collection are given. The french sequences investigated in the present study are marked in red.

**Figure S6: Phylogenetic analysis of S8 sequences of the EHDV-8 strains analyzed in this study.** Phylogenetic analysis of S8 sequences of EHDV-8 strains using the Maximum Likelihood method and Tamura-Nei model (1000 replicates). This analysis involved 34 nucleotide sequences; there were a total of 1128 positions in the final dataset. In the phylogenetic tree, GenBank sequences, species designations and strain names are given. Bootstrap values appeared at the corresponding nodes. In the phylogenetic tree, Genbank sequences, bluetongue serotype, country and year of sample collection are given. The french sequences investigated in the present study are marked in red.

**Figure S7: Phylogenetic analysis of S9 sequences of the EHDV-8 strains analyzed in this study.** Phylogenetic analysis of S9 sequences of EHDV-8 strains using the Maximum Likelihood method and Tamura-Nei model (1000 replicates). This analysis involved 33 nucleotide sequences; there were a total of 1094 positions in the final dataset. In the phylogenetic tree, GenBank sequences, species designations and strain names are given. Bootstrap values appeared at the corresponding nodes. In the phylogenetic tree, Genbank sequences, bluetongue serotype, country and year of sample collection are given. The french sequences investigated in the present study are marked in red.

**Figure S8: Phylogenetic analysis of S10 sequences of the EHDV-8 strains analyzed in this study.** Phylogenetic analysis of S10 sequences of EHDV-8 strains using the Maximum Likelihood method and Tamura-Nei model (1000 replicates). This analysis involved 34 nucleotide sequences; there were a total of 681 positions in the final dataset. In the phylogenetic tree, GenBank sequences, species designations and strain names are given. Bootstrap values appeared at the corresponding nodes. In the phylogenetic tree, Genbank sequences, bluetongue serotype, country and year of sample collection are given. The french sequences investigated in the present study are marked in red.
